# MAP kinase inhibitors stimulate T cell and anti-tumor activity in combination with blockade of the PD-L1/PD-1 interaction

**DOI:** 10.1186/2051-1426-1-S1-P79

**Published:** 2013-11-07

**Authors:** Bryan A Irving, Jeanne Cheung, Yagai Yang, Marina Moskalenka, Marcin Kowanetz, Heather Maecker, Ira Mellman

**Affiliations:** 1 Genentech, Inc., South San Francisco, CA, USA

## 

Pharmacological inhibition of the MAPK pathway with MEK or BRAF antagonists has proved successful in inducing regression of melanoma tumors bearing the targeted activating mutations. Moreover, antibodies targeting T-cell immune checkpoint inhibitors CTLA-4 or PD-L1/PD-1 have demonstrated the capacity to generate durable responses in patients with multiple cancer types. Thus, combining MAPK pathway-targeted agents with antibodies that enhance anti-tumor immunity represents an increasingly attractive treatment paradigm for cancer. However, little is known about the impact of tumor-targeted agents on immune function as similar signaling pathways drive both T-cell activation and cancer cell proliferation. Accordingly, agents targeting MAPK-dependent tumor growth would be predicted to also inhibit T-cell immunity. Here we show that, unexpectedly, potent suppression of T-cell receptor (TCR) function by MEK inhibition can be largely overcome in the presence of co-stimulation by anti-CD28 in vitro or blockade of the inhibitory PD-L1/PD-1 pathway in T cells in vivo. The ability of anti-CD28 to override suppression of T-cell activation by MEK inhibitors was dependent on the PI3K/mTOR pathway. Enhanced anti-tumor activity was also observed combining MEK inhibition with PD-L1 blockade, which was likely potentiated by upregulation of tumor MHC Class I expression through inhibition of MEK. Interestingly, inhibitors targeting BRAF V600E mutations actually augmented TCR-driven proliferation in vitro and T-cell function in vivo when combined with a vaccine or blockade of PD-L1 exclusively in the context of a wildtype BRAF background. These data demonstrate that targeting the MAPK pathway can be compatible with or even enhance T-cell function and provide rationale for combining these inhibitors with immunotherapy in clinical trials.

